# Establishment and Performance Evaluation of a Multiplexed TET2–APOBEC-Mediated cfDNA Methylation Detection Workflow Using qPCR and dPCR Readouts

**DOI:** 10.3390/jpm16050269

**Published:** 2026-05-18

**Authors:** Almudena Aguilera-Diaz, Philip B. Feinberg, Jianmin Huang, Eugene Spier, Francis Barany, Manny D. Bacolod

**Affiliations:** 1 Department of Microbiology and Immunology, Weill Cornell Medicine, New York, NY 10065, USA; ala4020@med.cornell.edu (A.A.-D.); pbf2001@med.cornell.edu (P.B.F.); jhuang@med.cornell.edu (J.H.); barany@med.cornell.edu (F.B.); 2 UniTaq Bio Inc., Los Altos, CA 94024, USA; gspier96@gmail.com

**Keywords:** enzymatic methylation conversion, cfDNA, TET2–APOBEC, colorectal cancer, qPCR, digital PCR, liquid biopsy, DNA methylation assay

## Abstract

**Background/Objectives**: Bisulfite-based cell-free DNA (cfDNA) methylation assays enable the detection of clinically valuable epigenetic biomarkers but often cause DNA degradation and inconsistent conversion efficiency, limiting performance in low-input liquid biopsy samples. We aimed to develop and evaluate a fully enzymatic cfDNA methylation workflow that preserves DNA integrity and supports quantitative clinical detection. **Methods**: The assay integrates TET2-mediated oxidation and APOBEC3A deamination with RNase H2-guided primer design, uracil-DNA glycosylase error suppression, and dual-probe detection compatible with quantitative PCR (qPCR) and digital PCR (dPCR). Performance was assessed using serial dilutions of methylated HT29 DNA, unmethylated controls, and plasma cfDNA from colorectal cancer (CRC) patients and healthy donors. Analytical sensitivity, linearity, and concordance between platforms were evaluated. **Results**: The 40-marker panel demonstrated higher cumulative methylation scores and more frequent methylation-positive signals in CRC cfDNA compared to controls. dPCR confirmed single-molecule resolution and clear discrimination between methylated and unmethylated templates, with occasional double-positive partitions consistent with mixed allelic methylation. Signal intensity across the dilution series followed a four-parameter logistic model, achieving detection sensitivity below 0.2% methylated DNA. qPCR and dPCR results showed strong correlation across the HT29 dilution series (R^2^ = 0.80) and high concordance in classifying CRC and healthy samples. **Conclusions**: This TET2–APOBEC-based enzymatic cfDNA assay enables sensitive, quantitative, sequencing-free methylation detection under gentle conditions, supporting its application in early colorectal cancer screening and routine clinical liquid biopsy workflows.

## 1. Introduction

Cell-free DNA (cfDNA) comprises short DNA fragments released into the bloodstream through apoptosis, necrosis, or active secretion by both normal and cancerous cells [1]. The tumor-derived fraction, circulating tumor DNA (ctDNA), contains genetic and epigenetic alterations that reflect the tumor’s molecular makeup. cfDNA analysis has therefore become a minimally invasive alternative to tissue biopsy, enabling long-term monitoring of tumor behavior, treatment response, and disease recurrence [1].

While mutation-based cfDNA assays have demonstrated clinical usefulness, their ability to detect early-stage disease is limited by the low levels of ctDNA. In contrast, DNA methylation changes occur early in tumor development, are specific to tissue types, and remain chemically stable in circulation [1,2]. Abnormal methylation of promoter CpG islands can silence tumor-suppressor genes, while global hypomethylation might activate cancer-driving pathways [1]. Therefore, cfDNA methylation analysis provides high biological specificity and reliable results, with multiple studies showing its superior ability for early detection and identifying the tissue of origin [3,4].

Over the last ten years, cfDNA methylation assays have evolved from being purely research tools to becoming diagnostic tests with clinical approval. The blood-based Epi proColon, a qPCR test that detects methylated SEPT9, was the first liquid biopsy approved by the FDA for colorectal cancer [5]. Similarly, stool-based Cologuard measures methylated NDRG4 and BMP3, along with mutation and hemoglobin markers [6]. Beyond colorectal cancer, methylation-based tests like HelioLiver (Helio Genomics) combine cfDNA methylation with protein biomarkers to detect hepatocellular carcinoma, achieving AUCs over 0.9 for early-stage detection [7]. Lung EpiCheck (Nucleix), a qPCR plasma test, identifies methylation changes associated with lung cancer, showing 85–90% sensitivity in prospective studies [8]. Sequencing-based tests such as Galleri (GRAIL), which pairs targeted methylation sequencing with machine learning [3,4], and Guardant Shield™, which exhibited 83% sensitivity and 90% specificity in the ECLIPSE trial [9], highlight the diverse clinical applications of cfDNA methylation analysis. However, despite these advances, most of these tests depend on bisulfite conversion—a crucial step that, although essential, introduces technical challenges that limit sensitivity and reproducibility.

Bisulfite conversion, introduced in the early 1990s, differentiates methylated from unmethylated cytosines through chemical sulfonation and deamination [10,11]. However, the harsh conditions of this reaction can degrade up to 84–96% of input DNA [12], a critical problem when working with scarce, fragmented cfDNA (~166–167 bp modal size) [13]. Incomplete conversion may yield false-positive signals, while strand breaks and over-conversion reduce amplification efficiency [14]. These issues collectively compromise assay performance and limit the practicality of bisulfite-based workflows in plasma-derived cfDNA testing.

To overcome these limitations, we developed an enzymatic cfDNA methylation assay that maintains DNA integrity while ensuring high conversion specificity. Instead of chemical deamination, it uses a two-step enzymatic process—oxidation of 5-methylcytosine by TET2 followed by deamination of unmethylated cytosines via APOBEC3A cytidine deaminase—enabling precise discrimination of methylated and unmethylated bases without degrading DNA [15]. The workflow is compatible with both qPCR and digital PCR (dPCR), with dPCR offering absolute quantification and excellent sensitivity for low-input samples [16,17].

This study evaluates the analytical performance of the enzymatic conversion workflow using model DNA and cfDNA from colorectal cancer cell lines (HT29), compares it with conventional bisulfite-based assays in experimental and clinical plasma samples, and assesses concordance between qPCR and dPCR readouts. By reducing DNA loss and simplifying workflow, this enzymatic method enables accurate cfDNA methylation analysis from small plasma volumes—addressing a key barrier to early cancer detection. Its compatibility with quantitative PCR platforms supports direct translation from research to clinical laboratories, establishing a next-generation framework for sensitive, reproducible, and clinically deployable cfDNA methylation diagnostics.

## 2. Materials and Methods

### 2.1. Preparation of DNA Templates

#### 2.1.1. Genomic DNA (gDNA) Was Extracted from the Human Colorectal Adenocarcinoma Cell Line HT29

The cells were cultured in McCoy’s 5A medium supplemented with 10% fetal bovine serum and 1% penicillin–streptomycin at 37 °C in a 5% CO_2_ incubator. When they reached 80–90% confluence, the cells were washed with phosphate-buffered saline and harvested by trypsinization, then centrifuged at 500× *g* for 5 min. The DNeasy Blood & Tissue Kit (Qiagen, Hilden, Germany ) was used to isolate the gDNA according to the manufacturer’s instructions. The extracted DNA was quantified with the Qubit 1X dsDNA High Sensitivity Assay Kit and Qubit 4.0 Fluorometer (Thermo Fisher Scientific, Carlsbad, CA, USA) and stored at −20 °C.

#### 2.1.2. Preparation of Methylation-Enriched DNA (Fully Methylated Control)

To create a fully methylated DNA reference, HT29 genomic DNA was enriched for methylation using the EpiMark Methylated DNA Enrichment Kit (New England Biolabs, Ipswich, MA, USA). This technique depends on the strong interaction between methylated CpG sites in double-stranded DNA and the methyl-CpG binding domain of MBD2 (MBD2-Fc) fused to the human IgG1 Fc region [18,19]. The fusion protein was attached to paramagnetic hydrophilic protein A/G beads, facilitating the specific capture of methylated DNA fragments. Enrichment was carried out following the manufacturer’s instructions, and the purified DNA was labeled as “fully methylated control DNA”.

#### 2.1.3. Whole-Genome Amplification of Unmethylated Reference DNA Was Performed

An unmethylated DNA control was prepared by whole-genome amplification (WGA) of pooled human genomic DNA from the buffy coat of 80 healthy donors, called “Roche DNA” (Roche Diagnostics, Basel, Switzerland). Multiple-displacement amplification [20] was carried out using the REPLI-g UltraFast Mini Kit (Qiagen, Hilden, Germany) following the manufacturer’s instructions to remove residual CpG methylation. The amplified product, named Whole-Genome-Amplified (WGA) DNA, was used as the unmethylated reference for later analyses.

#### 2.1.4. Preparation of Mixed DNA Templates to Simulate cfDNA Methylation Levels

To replicate the composition of circulating cell-free DNA (cfDNA) in clinical plasma—where tumor-derived DNA usually makes up only a small fraction of the total cfDNA [21]—fully methylated control DNA (HT29) was combined with unmethylated whole-genome-amplified (WGA) DNA from Roche in specific proportions. Mixtures were created so that the final methylated DNA concentrations were 0%, 0.156%, 0.312%, 1.25%, and 5% of the total genomic DNA. These standards served as models for cfDNA in subsequent assay development and sensitivity testing.

#### 2.1.5. Preparation of Cell-Free DNA (cfDNA) from Clinical Plasma Samples

Cell-free DNA was extracted from plasma specimens—four from colorectal cancer patients and four from healthy controls—obtained from Discovery Life Sciences (DLS) or Trans-Hit Bio (THB). The colorectal cancer samples included AD00074 (male, 30 years, stage III, DLS), AD00076 (male, 57 years, stage IV, DLS), AD00244 (female, 53 years, stage III, THB), and AD00319 (female, 52 years, stage III, THB). The healthy controls were AD00060 (female, 71 years, DLS), AD00266 (male, 44 years, THB), AD00334 (female, 66 years, THB), and AD00360 (female, 52 years, THB). All procedures adhered to Weill Cornell Medicine Institutional Review Board protocol #1308014272. Commercial biorepositories were instructed to follow a standardized plasma isolation protocol provided by our lab to ensure consistency and minimize genomic DNA contamination. cfDNA was isolated using the QIAsymphony SP automated system and the QIAsymphony DSP Circulating DNA Kit (Qiagen, Hilden, Germany), following the manufacturer’s instructions to maximize short cfDNA fragment recovery. The quality and size distribution of cfDNA were assessed with the Agilent 2100 Bioanalyzer using the High Sensitivity DNA Kit (Agilent Technologies, Santa Clara, CA, USA). DNA concentration was measured both from the Bioanalyzer electropherogram and independently quantified with the Qubit 1X dsDNA High Sensitivity Assay Kit and Qubit 4.0 Fluorometer (Thermo Fisher Scientific, Carlsbad, CA, USA).

### 2.2. Assay Design

**Figure 1:**
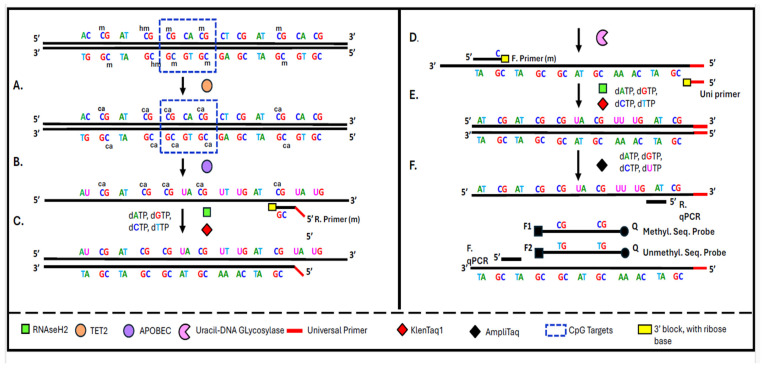
Schematic overview of the TET2–APOBEC enzymatic methylation assay for cfDNA. The workflow identifies CpG methylation markers associated with colorectal cancer (highlighted). TET2 first oxidizes 5-methylcytosine (mC) to carboxylcytosine (caC) (**A**), thereby protecting methylated cytosines from downstream deamination. In contrast, unmodified cytosines are selectively deaminated by the APOBEC enzyme into uracils (**B**). Uracil-DNA glycosylase removes uracil-containing strands, and RNase H2 cleaves any blocked primers, enabling strand-specific amplification (**C**–**E**). Targeted fragments are amplified with KlenTaq1 and a universal primer, and qPCR is used to differentiate methylated from unmethylated sites via sequence-specific probes (**F**). These steps align with Protocol A.

**Figure 2:**
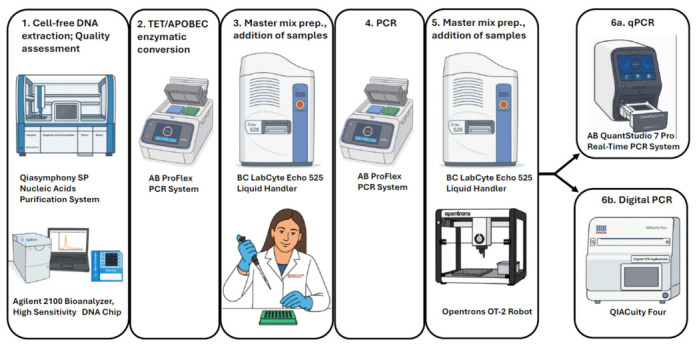
Instrumentation workflow for the cfDNA methylation assay. Cell-free DNA is extracted and quality-checked using the Qiagen QIAsymphony SP (Qiagen, Hilden, Germany) and the Agilent 2100 Bioanalyzer (Agilent, Santa Clara, CA, USA). It is then enzymatically converted using TET2/APOBEC enzymes on the ProFlex PCR system (Thermo Fisher Scientific, Waltham, MA, USA). Master mix preparation and sample addition are automated with liquid handlers such as the LabCyte Echo 525 (Danaher, San Jose, CA, USA) and Opentrons OT-2 (Opentrons, Brooklyn, NY, USA), followed by amplification on the ProFlex PCR system. Final results are measured either by qPCR using a QuantStudio 7 Pro (Thermo Fisher Scientific) or by digital PCR using a QIAcuity Four system (Thermo Fisher Scientific). Both Protocol A and Protocol B follow this same instrumentation workflow; the distinction between protocols lies in primer design and target amplification strategy rather than in instrumentation steps. The cartoon images included in Figure 2 were generated by ChatGPT-4o.

The CpG loci selected for the 40-marker methylation panel were chosen based on previous bioinformatic and statistical analyses detailed in our earlier publications [22,23]. All primers and probes used in the biochemical and instrumental procedures shown in Figure 1 and Figure 2 were designed using the PrimerQuest Tool (Integrated DNA Technologies, Coralville, IA, USA) Candidate sequences were evaluated for optimal melting temperatures, minimal secondary structures, and low cross-reactivity. All oligonucleotides were synthesized and purified by HPLC at IDT (Coralville, IA, USA).

### 2.3. Assay Protocol

#### 2.3.1. Enzymatic Conversion of DNA Templates (TET2–APOBEC System)

DNA templates from patient plasma cfDNA, methylated control mixes (HT29 genomic DNA diluted in unmethylated WGA DNA), and unmethylated control DNA (Roche WGA) were treated with the NEBNext Enzymatic Methyl-Seq Kit (E7120; New England Biolabs) for enzymatic cytosine conversion. This method used the TET2 enzyme to oxidize 5-methylcytosine (5mC) and 5-hydroxymethylcytosine (5hmC) into 5-formylcytosine (5fC) and 5-carboxycytosine (5caC). The oxidized bases became resistant to APOBEC (specifically APOBEC3A cytidine deaminase) deamination, while unmodified cytosines were deaminated to uracil and read as thymine during amplification, allowing single-base methylation calls without bisulfite-induced degradation [15]. After deamination, the reaction products were purified with NEBNext Sample Purification Beads to remove enzymes and buffer components. The DNA was eluted in 20 µL of elution buffer and transferred to DNA LoBind tubes (Eppendorf, Hamburg, Germany) for downstream analysis.

#### 2.3.2. Linear Amplification

Linear amplification was performed in 10 µL reactions containing: 2.0 µL of 5× GoTaq Flexi Buffer without Mg^2+^(Promega, Madison, WI, USA), 1.4 µL of 25 mM MgCl_2_, 0.2 µL of 10 mM dNTP mix, 0.25 µL of a 40-plex mixture of target-specific reverse primers (each with a 23 bp universal tail, 1 µM each), 0.2 µL of 5% Tween-20, 0.36 µL of RNase H2 (20 mU/µL; IDT), and 0.2 µL of Klentaq1 DNA polymerase (DNA Polymerase Technology, St. Louis, MO, USA) pre-mixed with Platinum Taq Antibody (Thermo Fisher Scientific) at a 1:10 ratio. Each reaction used 5 µL of TET2–APOBEC-converted DNA template, which was either 20–100 ng of cfDNA from plasma, 200 ng of the 5% HT29 mixture (positive control), or 1 µg of WGA Roche DNA (negative control). The use of a 20–100 ng input range reflects variability in cfDNA yield from clinical plasma samples and was chosen to ensure robust assay performance across a realistic range of input amounts typically encountered in liquid biopsy workflows. Amplification took place on a ProFlex PCR System (Thermo Fisher Scientific) with the following cycling program: 94 °C for 2 min; 20 cycles of 94 °C for 15 s, 60 °C for 80 s, and 72 °C for 30 s; then a final hold at 4 °C. Protocol A used reverse primers specific to methylated cfDNA templates, while Protocol B employed primers that amplified both methylated and unmethylated targets.

#### 2.3.3. Multiplex PCR Amplification

Linear amplification products (10 µL total) were divided into two 5 µL portions, each used as input for separate 20-plex PCR reactions. Each reaction used a unique set of 20 forward primers and a universal reverse primer that matches the tail sequence added during linear amplification. PCR was conducted in 10 µL mixtures containing 2.0 µL of 5× GoTaq Flexi Buffer, 1.4 µL of 25 mM MgCl_2_, 0.2 µL of 10 mM dNTPs, 0.2 µL of 5% Tween-20, 0.25 µL of the forward-primer mix (1 µM each), 0.4 µL of Antarctic Thermolabile UDG (1 U/µL; New England Biolabs), 0.145 µL of RNase H2 (100 mU/µL; IDT), 1.6 µL of Klentaq1 polymerase combined with Platinum Taq Antibody (1:10 ratio), 5.0 µL of the linear amplification product, and 0.1 µL of universal primer (100 µM). The thermocycling protocol on the ProFlex PCR System involved 37 °C for 10 min for UDG activity, then 28 cycles of 94 °C for 10 s, 60 °C for 80 s, and 72 °C for 30 s, concluding with a 4 °C hold. Protocols A and B used primer sets similar to those in the linear amplification step, designed for methylated-only and methylated-plus-unmethylated templates, respectively.

#### 2.3.4. Quantitative PCR (qPCR)

Quantitative PCR was performed for each CpG marker using single-plex reactions with a total volume of 5 µL. Each reaction included 2.25 µL of nuclease-free water (IDT), 2.5 µL of 2× TaqMan Fast Universal PCR Master Mix (Thermo Fisher Scientific), 0.05 µL of forward and reverse primers (100 µM each), 0.05 µL of FAM-labeled target and VIC-labeled reference probes (100 µM each), and 0.1 µL of PCR product. The reactions were run on a QuantStudio Pro 7 System (Thermo Fisher Scientific) with MicroAmp Fast 384-well plates sealed with optical adhesive film. The thermal cycling protocol involved 50 °C for 2 min, 95 °C for 20 s, followed by 45 cycles of 95 °C for 1 s and 60 °C for 20 s. Fluorescence data were collected in real time from FAM and VIC channels. For each of the 40 markers, threshold cycle (Ct) values were recorded for subsequent quantitative analysis.

#### 2.3.5. Digital PCR (dPCR)

Digital PCR reactions were conducted using the QIAcuity Digital PCR System (Qiagen). Each 13 µL reaction consisted of 9.7 µL of QIAcuity water, 3.0 µL of 4× Probe PCR Mix, 0.05 µL of each forward and reverse primers (100 µM), 0.05 µL of FAM-labeled target and VIC-labeled reference probes (100 µM), and 0.1 µL of diluted PCR product. The mixture was dispensed into a QIAcuity 96-well nanoplate, then automatically partitioned and amplified with the following program: 95 °C for 2 min; 40 cycles of 95 °C for 15 s and 60 °C for 30 s. Data collected after the run were analyzed using QIAcuity Suite Software to determine absolute copy numbers and methylation-specific signal distributions.

### 2.4. Analytical Performance Evaluation and Other Statistical Analyses

The analytical performance of the cfDNA TET2–APOBEC methylation assay was evaluated through a series of admixtures that mimic tumor-derived cfDNA fractions, and validation was performed on clinical plasma-derived cfDNA samples. Fully methylated HT29 genomic DNA was serially diluted into unmethylated whole-genome-amplified (WGA) DNA (Roche) to produce final methylated fractions of 0%, 0.156%, 0.312%, 1.25%, and 5%. Each mixture underwent the complete TET2–APOBEC enzymatic conversion and amplification process as described previously. Signal linearity, reproducibility, and concordance across platforms were assessed using quantitative PCR (qPCR) and digital PCR (dPCR). For qPCR, raw fluorescence data from FAM (methylated target) and VIC (reference/unmethylated control) channels were processed with QuantStudio Design and Analysis Software v2.6.0 (Thermo Fisher Scientific). Ct values were obtained using an adaptive baseline algorithm with manual adjustment as needed. Methylation signals were expressed as 40-Ct values, where higher values indicate stronger amplification. Digital PCR quantification of methylated and unmethylated templates was conducted using the QIAcuity system (Qiagen). Fluorescence data at the partition level were analyzed with QIAcuity Suite Software (v 3.1) to determine the number of FAM-positive (methylated) and VIC-positive (unmethylated) partitions [24]. The methylation percentage was then calculated as:

(1) Methylation Fraction = 100 × [FAM copies/(FAM copies + VIC copies)].

The same qPCR and dPCR techniques were employed to analyze clinical cfDNA samples from both colorectal cancer patients and healthy donors. All statistical tests, including correlation, concordance, and model fitting, were conducted with JMP Pro 17 (SAS Institute Inc., Cary, NC, USA). Graphs and regression results were generated in the same environment to visualize the relationships between qPCR and dPCR data. Heatmaps illustrating methylation signal distribution across the 40-marker panel were created using the web-based Morpheus platform (https://software.broadinstitute.org/morpheus/) (accessed on 20 September 2005).

## 3. Results

### 3.1. Serial Dilution Analysis Demonstrates Dose-Dependent and Reproducible Detection of Methylated DNA by the TET2–APOBEC Assay

**Figure 3:**
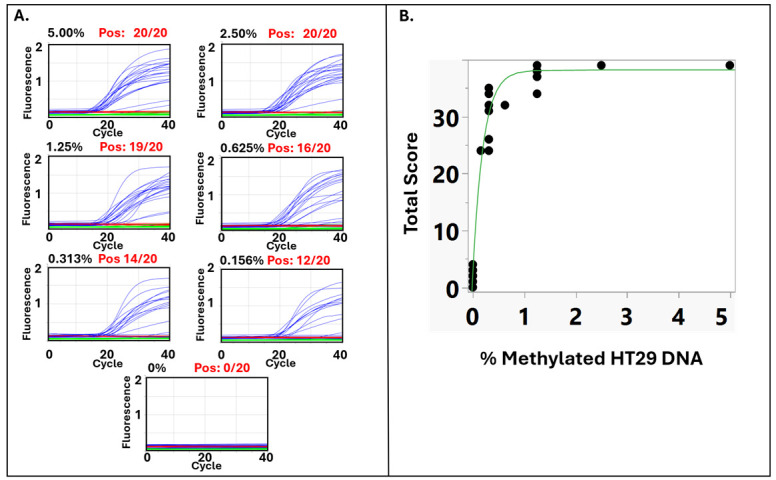
Performance analysis of the cfDNA TET2–APOBEC methylation assay with methylated HT29 DNA dilutions. (**A**) Representative qPCR amplification plots showing detection of methylated targets across serial dilutions of fully methylated HT29 genomic DNA (0–5%). Each panel displays the fraction of positively (Pos) detected methylation markers in the reaction plate. Blue curves indicate FAM (methylation) signals, green curves show VIC (wild-type/WT) signals, and red curves represent the Rox passive reference dye signals. (**B**) Logistic four-parameter model of the total methylation score (Class 1 score) versus the percentage of fully methylated HT29 DNA.

To assess the analytical performance of the cfDNA TET2–APOBEC methylation assay (Protocol A), serial dilutions of fully methylated HT29 genomic DNA were prepared, covering methylation fractions from 0% to 5%. Representative qPCR amplification plots (Figure 3A) demonstrated a clear dose-dependent increase in methylation signal with higher amounts of methylated DNA. All 20 CpG markers analyzed were detected positively (40 − Ct > 10) at input levels of 2.5% or higher (20/20), while fewer markers were positive within the 1.25% to 0.156% methylated DNA range. No amplification was observed in the 0% unmethylated control, confirming the assay’s high specificity and its ability to avoid false-positive signals from incomplete conversion or nonspecific amplification. The correlation between methylated DNA input and total methylation signal was modeled using a four-parameter logistic (4PL) regression (Figure 3B). The total methylation score (Class 1 score), defined as the sum of markers with 40 − Ct > 10, shows a sigmoidal relationship with increasing methylated DNA input, which aligns with the expected behavior of enzymatic amplification systems. The fitted 4PL curve fits the data well (R^2^ = 0.981), with the upper asymptote (d = 38.18; *p* < 0.0001) indicating the maximum assay response and the inflection point (b ≈ –2.63) representing the midpoint of sensitivity. Although the total methylation score is a discrete variable, the logistic model is suitable because the underlying detection process—enzymatic oxidation, deamination, and amplification of methylated templates—follows a continuous probabilistic response. Each marker contributes to an overall detection probability, so the total score transitions smoothly from low to high values as methylated DNA input increases. This pattern is analogous to classical enzyme or binding saturation behavior, in which signal increases nonlinearly as the fraction of reactive (methylated) targets rises before approaching a plateau [25]. In summary, these results show that the cfDNA TET2–APOBEC assay provides a reliable, dose-dependent, and biochemically consistent response to increasing methylated DNA input, supporting its quantitative accuracy and reproducibility in cfDNA methylation analysis.

### 3.2. cfDNA TET2–APOBEC Assay Reveals Tumor-Specific Methylation Signatures in Colorectal Cancer Plasma Samples

**Figure 4:**
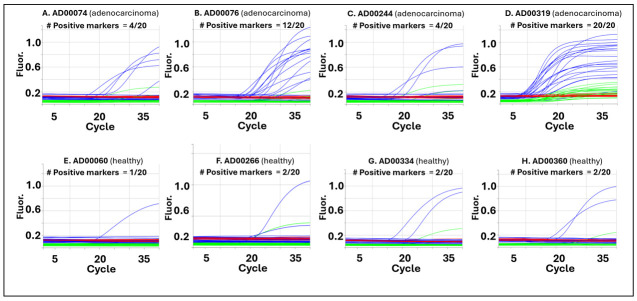
Representative qPCR amplification curves from the cfDNA 40-marker multiplex assay. Shown are amplification plots for four colorectal cancer (adenocarcinoma) cfDNA samples (**A**–**D**) and four healthy cfDNA samples (**E**–**H**). Each trace corresponds to one of the first 20 methylation markers included in the 40-marker multiplex panel. Blue curves represent FAM (methylation) signals, green curves represent VIC (wild-type/WT) signals, and red curves represent Rox passive reference dye signals. Colorectal cancer samples display robust amplification across multiple markers, consistent with tumor-specific methylation, whereas healthy cfDNA samples show little or no amplification. These results highlight the assay’s sensitivity for detecting cancer-derived methylation signals in plasma and demonstrate the feasibility of high-plex qPCR readout following TET2-APOBEC-based enzymatic conversion.

**Figure 5:**
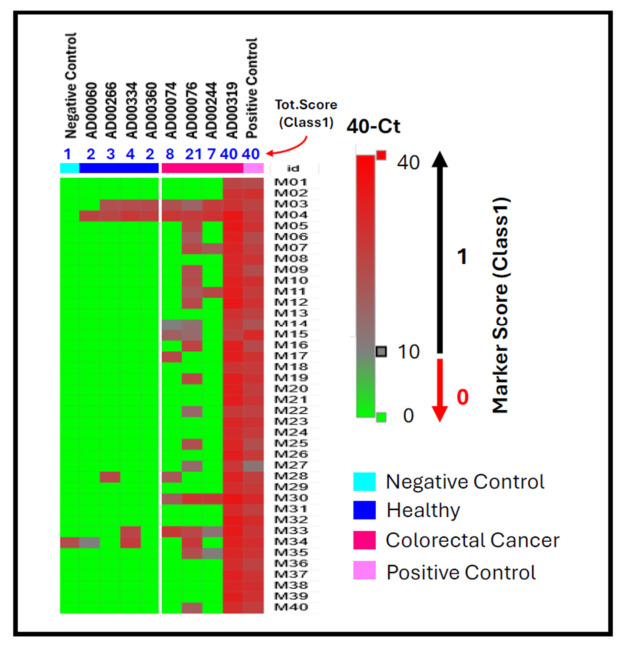
Heatmap of cfDNA methylation markers analyzed using the TET2–APOBEC assay (Protocol A). Shown are 40 CpG methylation markers analyzed in cfDNA from colorectal cancer patients, healthy donors, and positive/negative controls. Each cell represents the 40 − Ct value for a given marker (rows) and sample (columns), with color indicating methylation signal intensity (green = low, red = high). Blue numbers above the heatmap indicate the total Class 1 marker score (out of 40), corresponding to the number of markers with 40 − Ct > 10 for each sample. Colorectal cancer cfDNA samples exhibit higher total scores and more frequent methylation-positive markers than healthy and negative controls.

To evaluate whether the cfDNA TET2–APOBEC enzymatic workflow can detect tumor-associated methylation patterns in plasma, cfDNA from colorectal cancer (CRC) patients and healthy donors was analyzed using the 40-marker multiplex panel (Protocol A). Representative qPCR amplification plots (Figure 4) show that cfDNA from CRC patients (Panels A–D) exhibited strong amplification across multiple markers, while cfDNA from healthy donors (Panels E–H) showed minimal or no amplification. Each amplification trace represents one of the first 20 methylation markers in the multiplex panel. The earlier and more frequent amplification in CRC cfDNA samples highlights the assay’s sensitivity for detecting methylated cfDNA molecules at low abundance, consistent with tumor-derived methylation enrichment in these samples. A comprehensive comparison of all 40 markers is shown in Figure 5. The heatmap shows 40 − Ct values (rows = markers, columns = samples), with red indicating high methylation signals (40 − Ct > 10) and green showing low or no signals. Blue numbers above the heatmap represent the total Class 1 marker score—the number of methylation-positive markers per sample. Colorectal cancer cfDNA samples, such as AD00319 and AD00076, had the highest methylation burden, with total scores of 40 and 21, respectively, indicating widespread detection across nearly all markers. In contrast, healthy donor cfDNA samples like AD00060 and AD00266 showed very few positive markers, with total scores of only 2–3, while the negative control had a score of 1. The fully methylated HT29 DNA positive control yielded a total score of 40, confirming full panel detection and internal assay performance. These patterns highlight a clear difference in both per-marker methylation intensity and overall methylation scores between CRC and healthy samples. Although these differences are presented as exploratory observations due to the limited sample size, the consistent shift toward higher scores and broader methylation positivity in CRC cfDNA emphasizes the assay’s ability to distinguish tumor-derived methylation profiles from background cfDNA. This study primarily focuses on developing and validating the cfDNA TET2–APOBEC workflow. A larger clinical evaluation with an independent cohort of 216 plasma samples and machine-learning-based classification analysis is described in a separate manuscript. Together, these pilot data verify the assay’s reproducibility and demonstrate its potential for cfDNA-based cancer methylation detection.

### 3.3. Digital PCR Confirms Marker-Specific Methylation Differences Between Colorectal Cancer and Healthy cfDNA Samples

**Figure 6:**
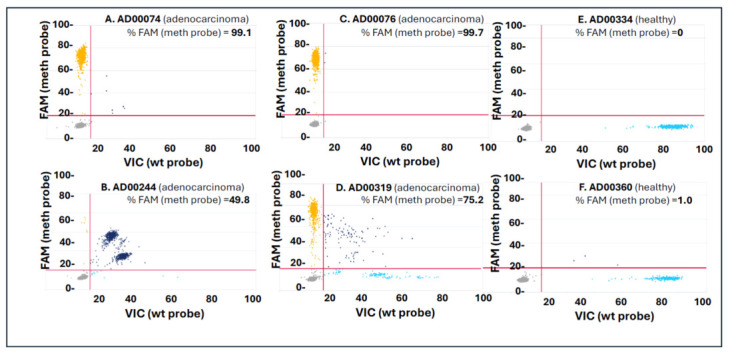
Digital PCR readout for Marker 30 using the QIAcuity system. Scatter plots show the distribution of partitions detected with methylation-specific FAM probes (*y*-axis) versus unmethylated wild-type VIC probes (*x*-axis). Red horizontal and vertical lines indicate the fluorescence thresholds for FAM and VIC signal classification, respectively. Gray dots represent double-negative partitions, orange dots represent FAM-positive methylated partitions, turquoise dots represent VIC-positive unmethylated partitions, and dark blue dots represent double-positive partitions containing co-detection of methylated and unmethylated templates within the same partition. Colorectal cancer cfDNA samples (**A**–**D**) exhibit higher fractions of FAM-positive partitions, while healthy cfDNA samples (**E**,**F**) predominantly show VIC-positive signals, consistent with the absence of methylation at this locus. Sample AD00244 (**B**) shows a substantial number of double-positive partitions, suggesting mixed allelic methylation. These results highlight the ability of digital PCR to resolve methylated versus unmethylated cfDNA at single-partition resolution and confirm locus-specific methylation differences between colorectal cancer and healthy plasma cfDNA.

To further validate methylation detection using an orthogonal quantitative platform, selected cfDNA samples from colorectal cancer (CRC) patients and healthy donors were analyzed with the QIAcuity digital PCR (dPCR) system. Figure 6 shows representative 2D scatter plots for Marker 30, which distinguish methylated from unmethylated cfDNA molecules based on probe-specific fluorescence. Each dot represents an individual partition (reaction well) classified by the presence of FAM signal (methylation-specific probe, *y*-axis) and VIC signal (wild-type/unmethylated probe, *x*-axis). The percentage of FAM-positive partitions (%FAM)—a measure of the methylated fraction—was calculated as: 100 × (Number of FAM-positive partitions)/(Total number of FAM + VIC-positive partitions). CRC cfDNA samples (Panels A–D) displayed significantly higher %FAM values (ranging from 49.8% to 99.9%) compared to healthy cfDNA samples (Panels E–F), which showed minimal methylation signals (%FAM ≤ 1%). These findings confirm the enrichment of tumor-derived methylation in CRC cfDNA and demonstrate the assay’s capability to distinguish methylation-positive targets at a single-molecule level. Notably, sample AD00244 (Panel B) exhibited a distinctive diagonal pattern, with many partitions testing positive for both FAM and VIC probes. This dual-probe positivity indicates the co-detection of methylated and unmethylated molecules within the same partition, likely due to the stochastic loading of both template types into a single droplet. Such double-positive (diagonal) partitions can be conceptualized as contributing roughly half of their counts to each probe channel (FAM and VIC), aligning with Poisson-based correction models used in digital PCR to interpret co-occupancy events [26]. Biologically, this pattern indicates mixed allelic methylation rather than true hemimethylation, reflecting partial methylation heterogeneity at this locus within the cfDNA population. In contrast, fully methylated CRC cfDNA samples, such as AD00074 and AD00076, showed partitions that were nearly entirely FAM-positive, consistent with a homogeneous methylation profile. Healthy donor cfDNA samples (e.g., AD00334 and AD00360) mostly displayed VIC-positive signals, indicating an unmethylated state at this marker. Overall, these digital PCR results support the qPCR findings, offering single-partition confirmation of methylation differences between CRC and healthy cfDNA. The high resolution of the QIAcuity system demonstrates that the cfDNA TET2–APOBEC workflow provides accurate, quantitative methylation detection and identifies locus-specific methylation heterogeneity in tumor-derived cfDNA.

### 3.4. Quantitative Performance of the TET2–APOBEC cfDNA Methylation Assay Under Digital PCR Detection

**Figure 7:**
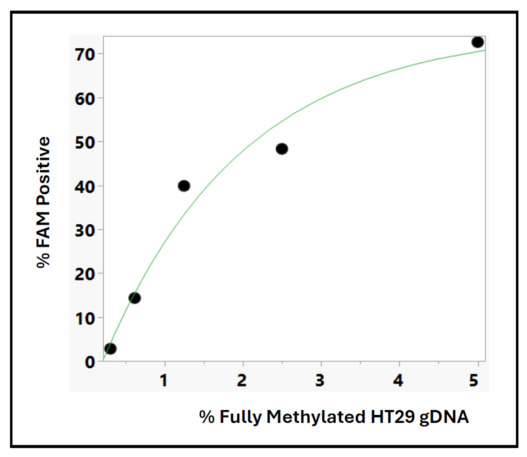
Digital PCR quantification of cfDNA methylation employs the TET2–APOBEC enzymatic workflow. Fully methylated HT29 genomic DNA was serially diluted with unmethylated DNA and analyzed for Marker 15 using the QIAcuity digital PCR platform. The *x*-axis indicates the percentage of methylated HT29 DNA, while the *y*-axis shows the percentage of FAM-positive partitions (%FAM). The relationship between methylated DNA input and %FAM displays a sigmoidal dose–response curve, which was modeled with a four-parameter logistic (4P) regression (green line), illustrating the assay’s quantitative capabilities in digital PCR detection.

Building on previous digital PCR (dPCR) analyses of clinical cfDNA samples, we further evaluated the quantitative response of the TET2–APOBEC cfDNA methylation assay using an in vitro DNA admixture model. Fully methylated HT29 genomic DNA was serially diluted with unmethylated DNA (0–5%) to simulate various levels of tumor-derived methylated cfDNA. After enzymatic conversion and amplification, the percentage of methylation-positive partitions (%FAM) was measured with the QIAcuity system (Figure 7). As expected, increasing amounts of methylated DNA led to higher FAM-positive rates, exhibiting a sigmoidal dose–response relationship between methylated DNA input and the %FAM signal. This pattern was accurately modeled by a four-parameter logistic (4P) regression, which effectively characterized the relationship between methylated DNA input and partition-level detection. The logistic curve represents the typical low detection threshold and saturation plateau seen in methylation assays, where detection probability rises nonlinearly as methylated templates become more abundant. Although %FAM is calculated from discrete partition counts, the 4P model is suitable because it captures the underlying enzymatic and hybridization kinetics that influence amplification efficiency in digital PCR. The steps involving TET oxidation and APOBEC deamination, followed by probe hybridization and polymerase extension, together produce a nonlinear yet consistent increase in amplification, aligning with the observed data. These findings overall confirm that the TET–APOBEC cfDNA methylation assay behaves quantitatively and reproducibly across a broad range of methylated DNA inputs under digital PCR conditions. The strong logistic fit supports the assay’s ability to accurately and proportionally measure methylated cfDNA and highlights its compatibility with digital detection platforms for translational and diagnostic use.

### 3.5. Concordance Between qPCR and Digital PCR Readouts Confirms Cross-Platform Reproducibility of the TET2–APOBEC Methylation Assay

**Figure 8:**
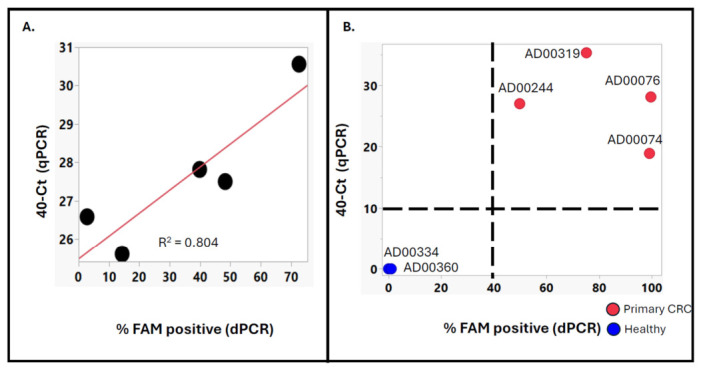
Concordance between qPCR and digital PCR readouts for cfDNA methylation markers. (**A**) Correlation between qPCR and digital PCR results for Marker 15, tested with a dilution series of fully methylated HT29 genomic DNA. The *x*-axis shows the percentage of FAM-positive partitions detected by digital PCR, and the *y*-axis displays average 40 − Ct values from qPCR. A strong positive correlation was observed, with Pearson R^2^ = 0.804. (**B**) Comparison of qPCR and digital PCR outcomes for Marker 30 in cfDNA from primary colorectal cancer (CRC) patients (red) and healthy donors (blue). All CRC samples fall in the quadrant where 40 − Ct > 10 and %FAM-positive > 40, while healthy samples cluster in the opposite quadrant (40 − Ct < 10 and %FAM-positive < 40), indicating consistent detection of methylation signals across both platforms.

To evaluate the cross-platform consistency of the cfDNA TET2–APOBEC methylation assay, we compared the outputs of qPCR and digital PCR (dPCR) for the same methylation markers using both in vitro standards and cfDNA from clinical plasma samples. Figure 8A illustrates the correlation between qPCR and dPCR results for serial dilutions of fully methylated HT29 genomic DNA. The *x*-axis shows the percentage of FAM-positive partitions detected by dPCR, while the *y*-axis displays the average 40 − Ct values obtained from qPCR. A strong positive correlation was observed (R^2^ = 0.804), indicating that increases in the proportion of methylated DNA detected by dPCR are closely reflected by earlier amplification signals (higher 40 − Ct) in qPCR. This agreement confirms that both platforms accurately quantify the same methylation dynamics across a range of input concentrations. Figure 8B expands this analysis to cfDNA samples from colorectal cancer (CRC) patients and healthy donors. For Marker 30, all CRC samples (red) clustered within the high-methylation quadrant defined by 40 − Ct > 10 and %FAM-positive > 40, while healthy controls (blue) fell into the opposite quadrant (40 − Ct < 10 and %FAM-positive < 40). This clear separation demonstrates consistent marker-specific methylation detection across both assay methods. The strong agreement between qPCR and dPCR highlights the robustness and reproducibility of the TET2–APOBEC methylation workflow, regardless of the detection platform used. qPCR offers a cost-effective, high-throughput screening method, while dPCR provides single-partition precision suitable for quantitative validation and low-abundance detection. Together, these two methods strengthen the assay’s analytical reliability, supporting its adaptability for both research and translational diagnostic applications. The strong agreement between qPCR and dPCR readouts further supports assay specificity, as both independent detection platforms consistently distinguish methylated from unmethylated templates across experimental conditions.

## 4. Discussion

We developed a fully enzymatic cfDNA methylation assay designed to achieve high analytical consistency through a simple, robust, and laboratory-compatible workflow. By integrating TET2–APOBEC enzymatic conversion [27] with probe-based amplification, methylation detection can be performed without bisulfite treatment or next-generation sequencing. Colorectal cancer (CRC) was selected as the initial model system due to its well-characterized cfDNA methylation landscape and the availability of validated CpG biomarkers for early detection. The assay produced consistent results on both qPCR and dPCR platforms, successfully differentiating colorectal cancer cfDNA from healthy plasma samples. These findings demonstrate that a sequencing-free, fully enzymatic workflow can match the analytical performance of more complex methylation profiling methods, making it suitable for routine diagnostics.

Traditional bisulfite-based methylation workflows often encounter challenges such as DNA degradation, high input requirements, and dependence on complex computational analysis. Bisulfite treatment causes DNA fragmentation and loss of cfDNA templates, restricting its clinical utility [12]. In contrast, the TET2–APOBEC enzymatic conversion used here gently oxidizes and deaminates cytosines under mild conditions, preserving cfDNA integrity and enabling analysis with nanogram-level inputs. Paired with RNase H2-dependent primer gating [28], which ensures amplification only when primers correctly hybridize, and the inclusion of uracil-DNA glycosylase [29] to remove incompletely converted bases, the workflow achieves high specificity and reproducibility. These biochemical refinements account for the clear separation of methylated (FAM) and unmethylated (VIC) partitions in dPCR, as well as the consistent correlation between expected and measured methylation levels. Overall, this enzymatic method significantly simplifies cfDNA methylation analysis, enabling the entire process—from cfDNA extraction to data interpretation—to be conducted using standard qPCR or dPCR instruments, without the need for library preparation, sequence alignment, or computational normalization.

Other bisulfite-free methods—such as TAPS (TET-assisted pyridine–borane sequencing) [30], ACE-seq (APOBEC-coupled epigenetic sequencing) [31], and EM-seq (enzymatic methyl-seq) [15,32,33]—allow cytosine conversion under mild, non-destructive conditions that maintain DNA integrity. In TAPS, 5-methylcytosine (5mC) and 5-hydroxymethylcytosine (5hmC) are oxidized by TET enzymes to 5-carboxylcytosine (5caC), which is then chemically reduced by pyridine–borane to dihydrouracil, which is read as thymine during sequencing. ACE-seq employs the cytidine deaminase APOBEC3A to convert unmodified cytosines to uracil while protecting 5mC and 5hmC through glucosylation, enabling base-specific discrimination after sequencing. EM-seq, which employs the same TET oxidation and APOBEC3A deamination principles as our platform, was developed for whole-genome or targeted next-generation sequencing. Although EM-seq and our enzymatic assay share the same core biochemistry, their detection strategies differ fundamentally. EM-seq infers methylation from base substitutions in sequencing reads, whereas our approach uses locus-specific probe-based amplification to directly distinguish methylated from unmethylated templates without sequencing. Collectively, these bisulfite-free methods have advanced methylation detection chemistry, yet most are still limited by their reliance on sequencing infrastructure.

While recent enzymatic workflows have produced excellent sequencing results on plasma cfDNA, they remain resource-intensive and reliant on deep sequencing and bioinformatic pipelines. Similarly, immunocapture [34] and nanopore-based methods provide valuable biological insights and, in the case of Oxford Nanopore Technologies (ONT) [35], offer a highly portable and rapidly evolving sequencing platform with strong translational potential. However, these methods currently rely on specialized instrumentation and signal-processing expertise. In contrast, our current platform converts enzymatic reactions into a self-contained, measurement-based assay that removes the need for sequencing entirely. This method fills a unique translational niche—offering locus-specific methylation analysis that clinicians can access directly, requires minimal computation, and is ideal for routine diagnostic use.

In addition, the workflow offers practical advantages in terms of cost and scalability. By eliminating the need for sequencing, library preparation, and extensive bioinformatic analysis, the assay can be implemented using widely available qPCR or dPCR platforms, reducing both operational complexity and overall cost compared to sequencing-based methylation approaches.

The strong linear correlation between expected and observed methylation fractions, along with the close agreement between qPCR and dPCR results, confirms the analytical reliability of the enzymatic cfDNA methylation platform. Although the workflow includes linear pre-amplification and multi-step PCR enrichment, the resulting signals remain proportional to input methylation ratios. For qPCR analysis, each marker’s cycle-threshold value is converted into a categorical score that indicates its methylation status, and these scores are summed to produce a total methylation score per sample. In our 216-sample cohort, the cumulative score displayed a clear stage-dependent trend—from normal through Stage I to Stage IV colorectal cancer—validated using ordinal regression analysis [36]. Eleven discrete marker-score categories captured progressive methylation changes across disease stages and served as inputs for downstream machine-learning models, which further improved classification accuracy. This integrated two-stage framework transforms qPCR and dPCR outputs into an interpretable, scalable diagnostic system that remains practical for clinical use. Across clinical cfDNA samples, the assay consistently produced methylation profiles that distinguished cancer from healthy controls, reflecting tumor-derived methylation signals in circulation. The standardized, streamlined analytical process aligns well with the operational needs of diagnostic laboratories, offering simplicity, rapid turnaround, and easy integration into existing data systems without requiring complex computational analysis.

We also observed variability in methylation signal intensity across the colorectal cancer cfDNA samples, most notably in sample AD00244, which had fewer methylation-positive markers. This variability likely reflects biological heterogeneity in tumor-derived cfDNA, including differences in tumor burden, ctDNA shedding rates, and underlying epigenetic profiles across patients. Technical factors, such as cfDNA input variability and fragmentation patterns, may also contribute. Given the limited sample size in this pilot analysis, these observations are exploratory; however, they are consistent with known inter-patient variability in cfDNA-based methylation assays and underscore the importance of evaluating this variability in larger clinical cohorts.

Building on this performance in colorectal cancer, the same enzymatic and analytical framework can be applied in other clinical contexts. Early detection efforts for lung [37], pancreatic [38], breast [39], and prostate [40] cancers have demonstrated the diagnostic promise of cfDNA methylation biomarkers. Several multi-cancer detection tests, such as Guardant Shield [9] and Grail’s Galleri [3], which employ targeted methylation signatures, are now in advanced stages of development. Methylation-based classifiers are also being explored as predictors of therapeutic response [41], including sensitivity to DNA-damaging agents [42] and immune checkpoint inhibitors [43]. Beyond oncology, cfDNA methylation analysis is gaining importance in other fields. In transplantation medicine, studies are examining graft-specific cfDNA methylation profiles as signs of rejection risk [44]. In neurological diseases, circulating methylated DNA fragments of neuronal origin are emerging as potential biomarkers for conditions such as Alzheimer’s disease [45], Parkinson’s disease [46], and epilepsy [47]. Collectively, these advances highlight the growing clinical relevance of cfDNA methylation analysis and underscore the value of simplified, sequencing-free workflows like the enzymatic platform described here, which can make such testing more accessible and scalable across disease areas.

The specificity of the TET2–APOBEC methylation assay is supported by several independent features of the workflow. First, unmethylated control samples consistently showed no detectable amplification, indicating minimal background signal. Second, strong concordance between qPCR and digital PCR platforms confirms that methylation-specific signals are reproducible and not assay-dependent artifacts. Third, probe-based detection, combined with RNase H2-mediated primer gating and UDG-based error suppression, ensures that only correctly converted and sequence-matched templates are amplified. Together, these design elements provide robust specificity without requiring additional orthogonal validation methods in this initial analytical study. Nevertheless, future studies may incorporate additional orthogonal validation approaches, such as sequencing-based or alternative methylation detection methods, to further confirm assay specificity across broader clinical settings.

Although our 40-CpG panel was optimized for colorectal cancer and evaluated in 216 clinical samples, larger and more diverse cohorts will be needed to confirm robustness and generalizability. Future multi-center studies should evaluate reproducibility across laboratories, sample types, and populations. Integrating into automated microfluidic dPCR systems may allow higher throughput, and combining with nanopore-based methylation calling could expand molecular coverage. These findings provide strong proof of concept for enzymatic cfDNA methylation detection in a sequencing-free, clinically adaptable format.

In summary, gentle enzymatic chemistry combined with standard amplification and signal-based detection creates a multiplex cfDNA methylation assay that is accurate, consistent, and easy to implement. By eliminating the need for bisulfite conversion, sequencing, and complex data analysis, this platform overcomes key technical and logistical challenges, paving the way for cfDNA methylation testing in routine clinical workflows. The same multiplex framework can be adapted for early cancer detection, recurrence monitoring, therapeutic guidance, and other disease applications, offering a flexible and scalable foundation for next-generation liquid-biopsy diagnostics.

## 5. Patent

A provisional patent application related to the assay and methods described in this manuscript has been submitted.

## Data Availability

The datasets generated during this study are not publicly available. Additional information may be obtained from the corresponding author upon reasonable request and subject to institutional review.

## Abbreviations

The following abbreviations are used in this manuscript:

TET2	Tet methylcytosine dioxygenase 2
APOBEC3A	Apolipoprotein B mRNA editing enzyme catalytic subunit 3A
dPCR	Digital PCR
cfDNA	Cell-free DNA
